# FilmArray™ GI panel performance for the diagnosis of acute gastroenteritis or hemorragic diarrhea

**DOI:** 10.1186/s12866-017-1018-2

**Published:** 2017-05-12

**Authors:** Antonio Piralla, Giovanna Lunghi, Gianluigi Ardissino, Alessia Girello, Marta Premoli, Erika Bava, Milena Arghittu, Maria Rosaria Colombo, Alessandra Cognetto, Patrizia Bono, Giulia Campanini, Piero Marone, Fausto Baldanti

**Affiliations:** 1 Microbiology and Virology Department, Fondazione IRCCS Policlinico San Matteo, Pavia, Italy; 2 Microbiology and Virology Unit, Fondazione Cà Granda Ospedale Maggiore Policlinico, Milan, Italy; 3 Center of HUS Control, Prevention and Management, Fondazione Cà Granda Ospedale Maggiore Policlinico, Milan, Italy; 4 Section of Microbiology, Department of Clinical, Surgical, Diagnostic and Pediatric Sciences, University of Pavia, Pavia, Italy

**Keywords:** Acute gastroenteritis, FilmArray™, Hemorragic diarrhea, Multiplex PCR

## Abstract

**Background:**

Acute gastroenteritis is a common cause of morbidity and mortality in humans worldwide. The rapid and specific identification of infectious agents is crucial for correct patient management. However, diagnosis of acute gastroenteritis is usually performed with diagnostic panels that include only a few pathogens. In the present bicentric study, the diagnostic value of FilmArray™ GI panels was assessed in unformed stool samples of patients with acute gastroenteritis and in a series of samples collected from pediatric patients with heamorragic diarrhea. The clinical performance of the FilmArray™ gastrointestinal (GI) panel was assessed in 168 stool samples collected from patients with either acute gastroenteritis or hemorragic diarrhea. Samples showing discordant results between FilmArray and routine methods were further analyzed with an additional assay.

**Results:**

Overall, the FilmArray™ GI panel detected at least one potential pathogen in 92/168 (54.8%) specimens. In 66/92 (71.8%) samples, only one pathogen was detected, while in 26/92 (28.2%) multiple pathogens were detected.

The most frequent pathogens were rotavirus 13.9% (22/168), *Campylobacter* 10.7% (18/168), *Clostridium difficile* 9.5% (16/168), and norovirus 8.9% (15/168). *Clostridium difficile* was identified only in patients with acute gastroenteritis (*p* < 0.01), while STEC was detected exclusively in patients with hemorragic diarrhea (*p* < 0.01). In addition, *Campylobacter spp., Salmonella spp.,* EPEC and *E. coli* producing Shiga-like toxin were more frequently detected in patients with hemorragic diarrhea (*p* < 0.05). The overall percent agreement calculated in samples was 73.8% and 65.5%, while 34.5% were discordant. After additional confirmatory analyses, the proportion of discordant samples decreased to 7.7%. Rotavirus and astrovirus were the most frequently unconfirmed pathogens.

**Conclusion:**

In conclusion, the FilmArray™ GI panel has proved to be a valuable new diagnostic tool for improving the diagnostic efficiency of GI pathogens.

## Background

Acute gastroenteritis (AGE) is a common cause of morbidity and mortality in humans worldwide [1]. The majority of cases occur in developing countries with poor hygiene standards and water sanitation problems. Acute gastroenteritis is the most severe and most common cause of diarrhea in children under 5 years of age [1]. Infectious gastrointestinal illness is a clinical syndrome whose aetiology is as varied as its presentation. Symptoms range from mild or self-limiting diarrhea to potentially life-threatening hemolytic uremic syndrome or pseudomembranous colitis. A wide range of pathogens cause acute gastroenteritis including viruses: norovirus, rotavirus and adenovirus [1], bacteria: *Campylobacter*, *Salmonella*, *Shigella*, *Escherichia*, and *Yersinia* species [2, 3]*,* and parasites: *Entamoeba hystolytica, Giardia,* and C*ryptosporidium* [4].

Hemolytic uremic syndrome (HUS) is the most common cause of pediatric acute kidney damage and is one of the most serious acute pediatric diseases with a fatality rate of 3% to 5% [5]. The disease, however, is not limited to children, as shown during an outbreak of Shiga toxin (Stx)–producing *Escherichia coli* (STEC) infection in Germany in 2011, which caused >800 adult cases [6]. In nearly 85% of cases, HUS develops as a complication of STEC intestinal infection with hemorragic diarrhea [7]. Diagnosis of STEC-HUS is currently based on the detection of Shiga toxins (Stx S) and/or isolation of STEC in stools.

The rapid and specific identification of infectious agents is crucial for appropriate patient management. In addition, surveillance of new cases is needed for outbreak prevention and control especially in close-contact communities such as hospitals and long-term care facilities. Unfortunately, the number of agents involved in gastrointestinal infections makes the construction of comprehensive diagnostic panels challenging. In fact, the diagnosis of acute gastroenteritis is usually either performed with diagnostic panels that include only a few pathogens or with diagnostic assays with limited performance [8, 9]. To overcome the difficulties in conventional gastroenteritis related diagnostics, a trend in recent years has been the introduction of molecular multiplex assays to replace and/or complement traditional microbiological tests [10–12]. The added value of molecular detection for enteropathogens in comparison with conventional methods has been demonstrated [13–16]. In this diagnostic context, the FilmArray™ technology (BioFire Diagnostics, Salt Lake City, Utah) has recently improved rapid PCR multiplexing. The FilmArray™ gastrointestinal (GI) panel was designed to simultaneously detect 22 of the most common gastrointestinal pathogens. The FilmArray GI panel offers high sensitivity and specificity [14, 17, 18] and has been recently used as point-of-care according to the syndromic approach [19].

In the present bicentric study, the diagnostic value of FilmArray™ GI panels was assessed in unformed stool samples of patients with AGE and in a series of samples collected from pediatric patients with heamorragic diarrhea.

## Methods

### Study population and samples

Unformed stool samples were retrospectively collected from patients with AGE from December 2014 through May 2015. The stool samples were stored at −80 °C and analyzed in June 2015 at the Microbiology and Virology Department, Fondazione IRCCS Policlinico San Matteo, Pavia (laboratory A) and Fondazione Cà Granda Ospedale Maggiore Policlinico, Milano (laboratory B). The latter is also a reference center for HUS control, prevention and management. Inclusion criteria were: i) hospitalization of patients with AGE; ii) hemorragic diarrhea in pediatric patients and iii) the availability of stool samples at GI syndrome onset. Exclusion criteria were: i) the presence of chronic diarrhea; ii) immunodeficiency of patients (transplant recipients and/or those undergoing chemotherapy); and iii) repeated samples.

This study was approved by the Institutional Review Board (IRB) of both centres. Informed consent was not required and samples were anonymized, only retaining gender, age and the category of clinical syndromes (acute gastroenteritis or hemorragic diarrhea) according to guidelines on the use of residual biological specimens for scientific purposes in keeping with Italian law (art.13 D.Lgs 196/2003).

### FilmArray™ GI panel

The following agents are included in the FilmArray™ GI panel (BioFire Diagnostics, Salt Lake City, UT): *Campylobacter* (*jejuni*, *coli*, and *upsaliensis*), *C. difficile* (Toxin A/B), *Plesiomonas shigelloides*, *Salmonella*, *Yersinia enterocolitica*, *Vibrio* (*parahaemolyticus*, *vulnificus*, and *cholerae*), *Vibrio cholera*, enteroaggregative *E. coli* (EAEC), enteropathogenic *E. coli* (EPEC), enterotoxigenic *E. coli* (ETEC), *Shiga*-like toxin-producing *E. coli* (STEC), *E. coli* O157, *Shigella*/enteroinvasive *E. coli* (EIEC), *Cryptosporidium* spp., *Cyclospora cayetanensis*, *Entamoeba histolytica*, *Giardia lamblia*, adenovirus (AdV) F40/41, astrovirus, norovirus GI/GII, rotavirus A, and sapovirus (I, II, IV, and V).

The FilmArray™ GI pouch system contains dried reagents for all the steps needed for extraction, PCR amplification and detection of the pathogens listed above. The pouch was rehydrated under negative pressure using the hydration injection vial. The correct volume of liquid was introduced into the pouch with a vacuum. Testing on the FilmArray™ platform (version 1.7) was performed according to the manufacturer’s instructions using 200 μl of stool re-suspended in Cary-Blair transport medium, which is the sample volume recommended by the manufacturer. Samples were diluted in sample buffer in the sample injection vial. The cannula of the sample injection vial was inserted into the pouch port and a pre-established volume of liquid was drawn into the pouch by vacuum. Results were available approximately 1 hour after placing the pouch in the FilmArray™ Instrument.

### Standard methods

At laboratory A, the stool samples were routinely tested for bacterial and parasitological pathogens using a combination of culture, immunochromatographic and molecular assays (Table 1). For virus detection, a panel of real-time RT-PCR or PCR detecting norovirus, astrovirus, rotavirus, adenovirus and sapovirus was performed as previously reported [20, 21].

**Table 1:** Methods routinely used in the two centers and additional assay used to confirm FilmArray GI results

Pathogen	Methods			
	Laboratory A^a^		Laboratory B^b^	
	In use	Analysis of discrepant results	In use	Analysis of discrepant results
*Clostridium difficile*	immunochromatographic test	Xpert^®^ *C. difficile/Epi* (real-time PCR)	Allplex™ GI assay	Xpert^®^ *C. difficile/Epi* (real-time PCR)
*Plesiomonas shigelloides*	none	none	Allplex™ GI assay	none
*Salmonella spp*	direct plating - culture	BD MAX™ Enteric Bacterial Panel	Allplex™ GI assay	BD MAX™ Enteric Bacterial Panel
*Yersinia enterocolitica*	direct plating - culture	none	Allplex™ GI assay	none
*Campylobacter spp. (jejuni, coli* and *upsaliensis)*	direct plating - culture	BD MAX™ Enteric Bacterial Panel	Allplex™ GI assay	BD MAX™ Enteric Bacterial Panel
*Vibrio spp. and V. cholerae*	direct plating - culture	none	Allplex™ GI assay	none
Enteroaggregative *E. coli* (EAEC)	direct plating - culture	none	Allplex™ GI assay	none
Enteropathogenic *E. coli* (EPEC)	direct plating - culture	none	Allplex™ GI assay	none
Enterotoxigenic *E. coli* (ETEC)	direct plating - culture	none	Allplex™ GI assay	none
*E.coli O157*	agglutination test	none	Allplex™ GI assay	none
Shiga-like toxin producing *E.coli* (STEC)	direct plating culture/immunochromatographic test	BD MAX™ Enteric Bacterial Panel	Allplex™ GI assay	BD MAX™ Enteric Bacterial Panel
*Shigella/*Enteroinvasive *E. coli* (EIEC)	direct plating - culture	BD MAX™ Enteric Bacterial Panel	Allplex™ GI assay	BD MAX™ Enteric Bacterial Panel
*Cryptosporidium*	direct microscopy/ immunochromatographic test	Allplex™ GI assay	direct microscopy/immunochromatographic test	Allplex™ GI assay
*Entameba histolytica*	direct microscopy/ immunochromatographic test	Allplex™ GI assay	direct microscopy/ immunochromatographic test	Allplex™ GI assay
*Cyclospora cayetanensis*	direct microscopy/ immunochromatographic test	Allplex™ GI assay	direct microscopy/ immunochromatographic test	Allplex™ GI assay
*Giardia lamblia*	direct microscopy/ immunochromatographic test	Allplex™ GI assay	direct microscopy/ immunochromatographic test	Allplex™ GI assay
Adenovirus,	real-time PCR (all AdV strains)	PCR/sequencing	FTD	PCR/sequencing
Rotavirus A,B	immunochromatographic test	real-time RT-PCR	FTD	Allplex™ GI assay
Astrovirus,	immunochromatographic test	real-time RT-PCR	FTD	Allplex™ GI assay
Sapovirus,	real-time RT-PCR	Allplex™ GI assay	FTD	Allplex™ GI assay
Norovirus	real-time RT-PCR	Allplex™ GI assay	FTD	Allplex™ GI assay

*NA* not available, *FTD* Fast-track Diagnostics

^a^Fondazione IRCCS Policlicnico San Matteo, Pavia

^b^Fondazione Cà Granda Ospedale Maggiore, Policlinico, Milano

At laboratory B, the Allplex™ GI one-step real-time RT-PCR assay (Seegene Inc., Seoul, South Korea) was used for the diagnosis of the following bacteria: *C. difficile* hypervirulent, *C. difficile* toxin B, *E. coli* O157, enterohemorrhagic *E. coli* (EHEC), enteropathogenic *E. coli* (EPEC), enterotoxigenic *E. coli* (ETEC), enteroaggregative *E. coli* (EAEC), *Campylobacter* spp., *Salmonella* spp., *Shigella* spp./EIEC, *Vibrio* spp. *Y. enterocolitica* and *Aeromonas* spp. Diagnosis of viruses was performed by using the Fast Track Diagnostic® (FTD®) viral gastroenteritis real-time RT-PCR kit (Fast Track Diagnostics, Luxemburg) in two tube multiplex plus add-on singleplex for the detection of norovirus G1 and G2, astrovirus, rotavirus, adenovirus, sapovirus and the internal control. For parasites, all stool samples were examined microscopically for the detection of ova, cysts and parasites.

### Assays for the analysis of discordant results

Samples showing discordant results between FilmArray and routine methods were further analyzed with an additional assay, where available (Table 1). Discordant bacteria-positive samples were tested by real-time PCR with the BD MAX™ Enteric Bacterial Panel (Becton Dickinson GmbH, Heidelberg, Germany), which detects *Salmonella spp.*, *Campylobacter spp. (C. jejuni and C. coli)*, *Shigella* spp./EIEC and STEC. Discordant samples positive for virus and parasites were re-tested with a specific real-time RT-PCR or the Allplex™ GI assay (mix 1, 4) including: Norovirus GIand GII, rotavirus, adenovirus, astrovirus, sapovirus, *G. lamblia*, *E. histolytica*, *Cryptosporidium* spp. and *C. cayetanensis* (Table 1).

### Criteria to resolve discrepant results

Results were considered true positives if: i) comparator testing and FilmArray™ were both positive (both true positive); ii) comparator testing was positive, FilmArray™ was negative and discrepancy analysis was positive (initial true positive, FilmArray™ false negative); and iii) comparator testing was negative, FilmArray™ was positive and discrepancy testing was positive (initial testing false negative, FilmArray™ true positive). On the contrary, results were considered true negatives if: i) initial testing and FilmArray™ were both negative (both true negative); ii) initial testing was negative, FilmArray™ was positive and discrepant testing was negative (initial testing true negative, FilmArray™ false positive); and iii) initial testing was positive, FilmArray™ was negative and discrepant testing was negative (initial testing false positive, FilmArray™ true negative).

### Statistical analyses

The categorical variables are given as numbers and percentages, and the between-group data were compared using contingency table analysis with the χ^2^or Fisher’s exact test, as appropriate. All of the analyses were two-tailed, and carried out using GraphPad Prism version 5 (GraphPad Software Inc., CA, USA); *p-*values of ≤0.05 were considered statistically significant.

## Results

### Patient characteristics

A total of 168 stool samples from as many patients (97 male and 71 female) were included in the study. Of these, 123/168 (73.2%) were patients with acute gastroenteritis (median age 16 years, range 1 month – 88 yrs) while 45/168 (26.8%) were children with hemorrhagic diarrhea (median age 3 years, range 2 months - 18 yrs). Among patients with acute gastroenteritis, 102/123 (82.9%) were hospitalized, 16/123 (13.0%) were seen in the emergency department and 5/123 (4.1%) were outpatients, while all 45 (100.0%) children with hemorrhagic diarrhea were hospitalized.

### FilmArray GI panel performance

Overall, the FilmArray™ GI panel detected at least one potential pathogen in 92/168 (54.8%) specimens, while 76/168 (45.2%) were negative. When considering positivity according to patient categories, we observed that 59/123 (47.9%) patients with acute gastroenteritis and 33/45 (73.3%) patients with hemorragic diarrhea were positive for at least one pathogen.

In 66/92 (71.7%) of the positive samples, only one pathogen was detected, compared to 14/92 (15.2%) with two pathogens, 10/92 (10.9%) with three and 2/92 (2.2%) with four pathogens. Of the single detections, bacteria were identified in 34/66 (51.5%) samples, compared to viruses in 24/66 (36.4%) samples and parasites in 8/66 (12.1%) cases. Of the multiple detections, a wide range of combinations was observed: two bacteria and one virus (7/26; 26.9%), three bacteria (6/26; 23.1%), and one bacteria and one virus (6/26; 23.1%), which proved to be the most frequent (data not shown). The most prevalent pathogens were rotavirus 13.9% (22/168), *Campylobacter* spp. 10.7% (18/168), *C. difficile* 9.5% (16/168), norovirus 8.9% (15/168), *Salmonella* spp. 7.1% (12/168), EPEC 6.0% (10/168), STEC 4.2% (7/168), EAEC 2.9% (5/168), *G. lamblia* 2.4% (4/168), sapovirus 2.4% (4/168), ETEC 1.8% (3/168), *E. histolytica* 1.8% (3/168)*,* astrovirus 1.8% (3/168), *Shigella*/EIEC 1.8% (3/168), *Cryptosporidium* 1.2% (2/168), *Y. enterocolitica* 0.6% (1/168) and adenovirus 0.6% (1/168). No positive samples for *P. shigelloides, Vibrio spp.* and *C. cayetanensis* were found. The great majority of pathogens were identified in both single and multiple infections, while EPEC (10/168, 6.0%), ETEC (3/168,1.8%), *Y. enterocolitica* (1/168, 0.6%) and adenovirus (1/168, 0.6%) were observed only in co-infections with at least one other pathogen.

**Fig. 1:**
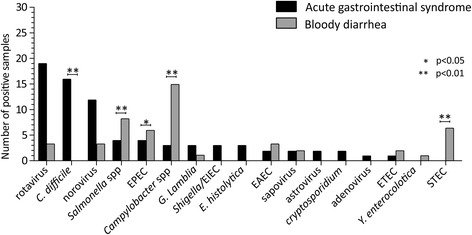
Distribution of pathogens detected by the FilmArray™ GI panel according to patient category

As shown in Fig. 1, the pattern of pathogens detected in patients with AGE and hemorragic diarrhea was significantly different. Specifically, *C. difficile* was detected exclusively in patients with AGE (16 vs 0; *p* < 0.01), while STEC was detected exclusively in patients with hemorragic diarrhea (6 vs 0; *p* < 0.01). *Campylobacter spp., Salmonella* spp.*,* and *E.coli* EPEC were more frequently detected in patients with hemorragic diarrhea (*p* < 0.05). To sum up, the overall percent agreement calculated was 73.8%, while the positive and negative percent agreements were 87.5% and 77.1%, respectively.

### FilmArray™ GI vs comparators

Of the positive FilmArray™ results (*n* = 92), 50/92 (54.3%) were concordant with the initial results, while 42/92 (45.7%) results were discordant (Table 2). In 22 out of 42 (52.4%) discordant samples, FilmArray™ identified at least one additional pathogen (Table 2 samples #1–22). After analysis of the discrepant results, the FilmArray™ results were confirmed in 17/22 (77.3%) samples, whereas in 5/22 (22.7%) samples, additional pathogens (three rotaviruses, one astroviruses and one sapovirus) identified by FilmArray™ were not confirmed. In addition, five pathogens remained unidentified due to the lack of confirmatory tests for *Cryptosporidium*, EPEC and EAEC. In 7/42 (16.3%) discordant samples, FilmArray™ failed to detect at least one additional pathogen (Table 2, samples #23–29). In 4/7 (57.1%) of these samples, FilmArray™ results were confirmed using the additional methods, while in 1/7 (14.3%) sample (#23) FilmArray™ results was not confirmed. However, the identified AdV strain was different from those included in the GI panel (F40/41). In 2/7 (28.6%) cases, no additional test was available to confirm EPEC detection. Finally, in the remaining 13/42 (30.2%) discordant samples, FilmArray™ identified at least one additional pathogen but failed to detect at least one other pathogen (Table 2, samples #30–42). In 6/13 (46.2%) samples, the FilmArray™ results were confirmed. In 2/13 (15.4%) samples, FilmArray™ results were not confirmed and thus false positive results were observed for one Shigella/Enteroinvasive E. coli and one rotavirus, whereas a false negative result was observed for one adenovirus (typed as AdV-C1). Due to the lack of confirmatory tests for EPEC, EAEC, 4/13 (30.8%) cases remained unresolved. Finally, in sample #40, the Allplex™ GI assay detected *C. difficile*, while FilmArray™ GI detected *Salmonella spp.*, norovirus and rotavirus. Negative results were obtained using the Allplex™ GI as a comparator method for viruses, and the BD MAX™ Enteric Bacterial Panel for bacteria. Overall, in 28/42 cases (66.6%), the FilmArray™ results were confirmed after analysis of discrepant results.

**Table 2:** Analysis of discrepant results in FilmArray™ GI positive samples

#	Lab	Cat.	Laboratory test results^a^	FilmArray™ results^a^	Discrepancy analysis assays	Case resolution	Interpretation of discordance	Final analysis of FilmArray™ results
1	A	AGE	N	***Shig*** **/EIEC, ETEC**	BD MAX™ Enteric Bacterial Panel (*shig/*EIEC)	*Shig*/EIEC, ETEC	TP	+
2	A	AGE	N	***C. difficile***	Xpert^®^ *C. difficile/Epi* (real-time PCR)	*C. difficile*.	TP	+
3	A	AGE	rotavirus	rotavirus, ***C. difficile***	Xpert^®^ *C. difficile/Epi* (real-time PCR)	rotavirus, *C. difficile*	TP	+
4	A	AGE	N	**rotavirus**	real-time RT-PCR	rotavirus	TP	+
5	A	AGE	N	***C. difficile***	Xpert^®^ *C. difficile/Epi* (real-time PCR)	*C. difficile*	TP	+
6	A	AGE	*G. lamblia*	*G. lamblia* ***, shig/*** **EIEC, EAEC**	BD MAX™ Enteric Bacterial Panel (*shig/*EIEC)	*G. lamblia*, shig/EIEC, EAEC	TP	+
7	A	AGE	N	**rotavirus**	real-time RT-PCR	N	TN	FP (rotavirus)
8	A	AGE	N	**rotavirus**	real-time RT-PCR	N	TN	FP (rotavirus)
9	A	AGE	N	**cryptosporidium**	none	NA	NA	NA
10	A	AGE	*C. difficile*	*C. difficile* **, astrovirus**	Real-time RT-PCR	*C. difficile*	TP	+
11	A	AGE	N	**astrovirus**	Real-time RT-PCR	N	TP	+
12	A	AGE	*C. difficile*, rotavirus	*C. difficile*, rotavirus, **EPEC**	NA	*C. difficile*, rotavirus	NA (EPEC)	NA
13	B	AGE	*Camp. spp.*, rotavirus	*Camp. spp.*, rotavirus, ***shig/*** **EIEC**	BD MAX™ Enteric Bacterial Panel	*Camp. spp.*, rotavirus, *Shig/*EIEC	TP	+
14	B	AGE	adenovirus	adenovirus**, sapovirus**	Allplex™ GI assay	adenovirus, sapovirus	TP	+
15	B	AGE	norovirus	norovirus, ***salmonella*** **, rotavirus**	BD MAX™ Enteric Bacterial Panel (*Salmonella*) Allplex™ GI assay (rotavirus)	norovirus, *Salmonella spp*	TP (*Salmonella spp)* TN	**+** FP (rotavirus)
16	B	HD	*Salmonella spp.*	*Salmonella spp.,* **EPEC, norovirus**	Allplex™ GI assay	*Salmonella spp.*, norovirus	NA (EPEC) TP (norovirus)	NA **+**
17	B	HD	*Camp. spp*	*Camp. spp.*, **EPEC**	None	*Camp. spp*	NA (EPEC)	NA
18	B	HD	N	**rotavirus**	real-time PCR	N	TP	+
19	B	HD	N	**rotavirus**	real-time PCR	N	TP	+
20	B	HD	N	**EAEC**	none	NA	NA	NA
21	B	HD	*Salmonella spp.*	*Salmonella spp.,* **astrovirus, EAEC**	Allplex™ GI assay (astrovirus) none (EAEC)	*Salmonella spp*	TN NA (EAEC)	FP (astrovirus) NA
22	B	HD	EHEC, EAEC, ETEC, EPEC	STEC, EAEC, ETEC, **sapovirus**	Allplex™ GI assay (sapovirus) none (EPEC)	STEC, EAEA	TN (sapovirus) NA	FP (sapovirus) NA
23	B	AGE	rotavirus, **adenovirus**	rotavirus	PCR and sequencing	rotavirus, adenovirus (AdV-C1)	TP (adenovirus)	+^b^ (adenovirus)
24	B	HD	*Camp. spp.,* ***Shigella spp***	*Camp. spp*	BD MAX™ Enteric Bacterial Panel	*Camp. spp*	TN	+
25	B	HD	*Camp. spp.,* **EPEC**	*Camp. spp*	none	*Camp. spp*	NA	NA
26	B	HD	*Camp. spp.,* ***Shigella spp***	*Camp. spp*	BD MAX™ Enteric Bacterial Panel	*Camp. spp*	TN	+
27	B	HD	*Camp. spp.,* ***Shigella spp***	*Camp. spp*	BD MAX™ Enteric Bacterial Panel	*Camp. spp*	TN	+
28	B	HD	STEC, *Y. enterocolitica,* ***C. difficile***	STEC, *Y. enterocolitica*	BD MAX™ Enteric Bacterial Panel	STEC, *Y. enterocolitica*	TN (*C. difficile*)	+
29	B	HD	STEC, **EPEC**	STEC	none	STEC	NA (EPEC)	+
30	A	AGE	norovirus, ***C. difficile***	norovirus, **EPEC**	Xpert^®^ *C. difficile/Epi* (real-time PCR) NA (EPEC)	norovirus	TN (*C. difficile*) NA (EPEC)	+ NA
31	A	AGE	***C. difficile***	***Camp. spp***	Xpert^®^ *C. difficile/Epi* (real-time PCR) BD MAX™ Enteric Bacterial Panel *(Camp spp)*	*Camp.* spp	TN (*C. difficile*) TP (*Camp. spp.*)	+ +
32	A	AGE	**rotavirus**	***Camp. spp***	real-time PCR (rotavirus) BD MAX™ Enteric Bacterial Panel *(Camp spp)*	*Camp.* spp	TN (rotavirus) TP (*Camp. spp.*)	+ +
33	A	AGE	rotavirus, **adenovirus**	rotavirus, **EPEC**	PCR/sequencing (adenovirus) EPEC (NA)	adenovirus (AdV-A12)	TN (adenovirus)^b^ NA(EPEC)	+^b^ NA
34	B	AGE	norovirus, *salmonella spp.*, **adenovirus**	norovirus, *salmonella spp.*, **rotavirus**	Allplex™ GI assay	norovirus, *salmonella spp*	TN (adenovirus) TN	+ FP (rotavirus)
35	B	AGE	**norovirus**	**sapovirus**	Allplex™ GI assay	sapovirus	TP (sapovirus) TN (norovirus)	+ +
36	B	AGE	**adenovirs**	**Shig/EIEC**	BD MAX™ Enteric Bacterial Panel (Shig/EIEC) Allplex™ GI assay (adenovirus)	adenovirus (AdV-C1)	TN (*Shig/EIEC*) TP (adenovirus)^b^	FP (shig/EIEC) +^b^ (adenovirus)
37	B	HD	sapovirus**,** EPEC**, STEC**	sapovirus*,* EPEC, ***Camp. spp.*** **,**	BD MAX™ Enteric Bacterial Panel	*Camp. spp*	TN (STEC) TP (*Camp. spp.*)	+ +
38	B	HD	***C. difficile***	***Salmonella spp***	BD MAX™ Enteric Bacterial Panel	*Salmonella spp*	TN (*C. difficile*) TP (*Salmonella spp*)	+ +
39	B	HD	***C. difficile***	***Salmonella spp.,*** **rotavirus, norovirus**	BD MAX™ Enteric Bacterial Panel	Negative with confirmed test	NA	NA
40	B	HD	**EPEC**	**STEC** ***, G. lamblia***	BD MAX™ Enteric Bacterial Panel	*STEC*, *G. lamblia*	TP (*Stx1/2*) TP (*G. lamblia*)	+ +
41	B	HD	**E. coli O157**	**EPEC, ETEC**	BD MAX™ Enteric Bacterial Panel	negative	NA	NA
42	B	HD	**EAEC**	**STEC**	BD MAX™ Enteric Bacterial Panel	STEC	TP (STEC) NA (EAEC)	+ NA

*N* negative, *ND* not done, *NA* not applicable, *FTD* fast-track diagnostic, *AGE* acute gastroenteritis, *HD* hemorragic diarrhea

Pathogens analyzed with additional assays are reported in bold. No confirmatory assays were available for *Plesiomonas shigelloides, Yersinia enterocolitica, Vibrio spp.*, EAEC, EPEC ETEC and E. coli O157

^*a*^The pathogens included in the discrepancy analysis are reported in bold.

^*b*^adenovirus strains different from F40–41 were not included in the FilmArray™ GI Panel

Of the FilmArray™ negative results (*n* = 76), 60/76 (78.9%) were concordant with the initial routine testing results, while discordant results were observed in 16/76 (21.1%) samples. In 9/16 (56.1%) discordant samples an adenovirus was detected, compared to a rotavirus in 4/16 (25.0%) samples, *C. difficile* in 1/16 (6.3%), an *E. histolytica* in 1/16 (6.3%), and an *aeromonas* in 1/16 (6.3%) samples*.* Results from alternative assays are reported in Table 3. All nine samples that were positive for adenovirus were sequenced and in 8/9 cases, the AdV strains (three AdV-A12, three AdV-C1, one AdV-C1 and one AdV-C2) were different from those included in the GI panel (F40/41). Sequencing failed in one sample and it was therefore excluded from the number of discordant samples. None of the rotavirus-positive samples were confirmed with the additional real-time assay. Finally, the sample that was positive for *aeromonas* was confirmed as positive but, *aeromonas* was not included in the FilmArray™ GI panel and therefore this result could not be considered as truly discordant. Overall, 10/16 (62.5%) results were confirmed as truly discordant.

**Table 3:** Results of discrepancy analysis in FilmArray™ GI negative samples

#	Lab	Cat.	Laboratory test results	FilmArray™ results	Discrepancy analysis assays	Case resolution	Interpretation of discordance	Final analysis of FilmArray™ results
1	A	AGE	rotavirus	N	real-time RT-PCR	N	TN	+
2	A	AGE	rotavirus	N	real-time RT-PCR	N	TN	+
3	A	AGE	*C. difficile*	N	Xpert^®^ *C. difficile/Epi* (real-time PCR)	*C. difficile*	TP	FN (*C. difficile)*
4	A	AGE	adenovirus	N	PCR/sequencing	adenovirus type A12	TP^a^	+^a^ (adenovirus)
5	A	AGE	*E. histolytica*	N	Allplex™ GI assay	*E. histolytica*	TP	FN (*E. histolytica)*
6	A	AGE	rotavirus	N	real-time RT-PCR	N	TN	+
7	A	AGE	adenovirus	N	sequencing	adenovirus type A12	TP^a^	+^a^ (adenovirus)
8	A	AGE	adenovirus	N	sequencing	adenovirus type A12	TP^a^	+^a^ (adenovirus)
9	A	AGE	rotavirus	N	real-time RT-PCR	N	TN	+
10	B	AGE	adenovirus	N	PCR/sequencing	adenovirus type C1^a^	TP^a^	+^a^ (adenovirus)
11	B	AGE	adenovirus	N	PCR/sequencing	adenovirus type C5^a^	TP^a^	+^a^ (adenovirus)
12	B	AGE	adenovirus	N	PCR/sequencing	P not typed	TP^a^	NA
13	B	AGE	adenovirus	N	PCR/sequencing	adenovirus type C1^a^	TP^a^	+^a^ (adenovirus)
14	B	AGE	adenovirus	N	PCR/sequencing	adenovirus type C2^a^	TP^a^	+^a^ (adenovirus)
15	B	AGE	adenovirus	N	PCR/sequencing	adenovirus type C1^a^	TP^a^	+^a^ (adenovirus)
16	B	HD	*aeromonas* ^*a*^	N	Allplex™ GI assay	*aeromonas*	TP^a^	NA

*FTD* fast-track diagnostics, *TP* true positive, *TN* true negative, *FN* false negative, *NA* not applicable, *AGE* acute gastroenteritis, *HD* hemorragic diarrhea. No confirmatory assays were available for *Plesiomonas shigelloides, Yersinia enterocolitica, Vibrio spp.*, EAEC, EPEC ETEC and E. coli O157

^*a*^ *aromonas* and adenovirus types different from F40–41 were not included in the FilmArray™ GI Panel

## Discussion

In the present study, the FilmArray™ GI panel was evaluated in a series of unformed stool samples of patients with GI syndrome and pediatric patients with hemorragic diarrhea. Overall, the FilmArray™ GI panel identified a pathogen in at least 50% of analyzed samples. Of the patients with hemorragic diarrhea, the percentage reached 70%. Overall, the detection rate observed in the FilmArray™ analyses ranged from 33.0% to 62.7% [14, 18, 22–25]. This wide range of detection frequencies could be attributed to the different patient categories analyzed (adult vs pediatric or outpatient vs inpatient). Other FilmArray™ studies analyzing patient populations similar to those included in our study showed a nearly identical positivity rate [18, 23]. On the contrary, in studies where all or the great majority were patients examined in the outpatient setting, the frequency of detection was lower (32.9% and 40.4%) than studies analyzing hospitalized patients [24, 25]. Our data are also in keeping with a multicenter European study performed with the FilmArray™ GI panel aimed at determining the spectrum of possible pathogens involved in acute community-acquired gastroenteritis [22].

Of the positive samples, as expected, the more prevalent pathogens were rotavirus, *C. difficile,* norovirus and *Salmonella* spp., as also observed by others [14, 22, 26, 27]. The FilmArray™ GI panel detected a series of diarrheagenic *E.coli* (DEC) isolates (i.e. EPEC, ETEC, EAEC, EPEC and *shig*/EIEC), which were not included in the routine laboratory procedures for one of the two centers. For many years, these E.coli strains have been considered a leading cause of gastroenteritis only in developing countries [28]. However, there is also a growing number of reports on this problem in developed countries [29, 30]. In addition, DEC types have frequently been observed in co-infections with other enteropathogens with increased illness severity, especially in mixed infections with rotavirus [30]. Nevertheless, in our study, EPEC and ETEC were detected only in coinfections, as also previously observed [25]. Thus, the clinical relevance of these pathogenic *E.coli* need to be fully elucidated with a case-control study including also asymptomatic patients.

Interesting results were obtained when the distribution of pathogens was analyzed according to the patient category. In patients with acute gastroenteritis, rotavirus and norovirus were the main pathogens detected, along with a significant number of *C. difficile.* In contrast, no patients with hemorragic diarrhea tested positive for *C. difficile*. Indeed, there is very limited published data on *C. difficile* associated with hemorragic diarrhea and only sporadic cases have been reported [31–33]. On the other hand, in patients with hemorragic diarrhea, *Campylobacter* spp., *Salmonella* spp., EPEC and STEC (non-O157) were the main agents detected. Regarding the etiology of HUS, STEC is the most common reported cause of this syndrome in children and this association (HUS-STEC) is now under epidemiological surveillance in the EU [34, 35]. Some older reports have described an association between *Campylobacter* spp. and HUS [36, 37]. More recently, a meta-analysis on the proportion of Campylobacter that develops chronic sequelae (i.e. HUS) was estimated to be lower than 0.01% [38]. On the contrary, our observations are in keeping with a recent Italian clinical report testing 1251 patients, where *Campylobacter* spp. and *Salmonella* spp. were also identified with an unexpectedly high frequency [39]. The authors suggested that the synergic activity played by *Campylobacter* and *Salmonella* infection contributed to STEC and EPEC infection.

The data from this evaluation demonstrated that in about 35% of samples, discordant results were observed when comparing the diagnostic procedures of the two laboratories. In the vast majority, the FilmArray™ GI panel identified additional pathogens. It is worth noting that an initial increase in the detection of adenovirus was observed in samples under investigation. However, only a few adenovirus-positive samples were confirmed as type F40–41 by molecular typing. Although the performance of the FilmArray™ GI panel in the identification of adenovirus commonly associated with gastroenteritis (i.e. F40/41) proved satisfactory, the FilmArray™ GI panel missed a series of species C and A adenoviruses detected in unformed stools by other methods [40, 41]. The clinical impact of these “atypical” gastroenteric adenoviruses should be further investigated to provide evidence of their clinical significance in gastroenteric syndrome. Overall, after additional confirmatory analyses, the proportion of discordant samples decreased to 7.7%. Rotavirus and astrovirus were the most frequently unconfirmed pathogens. The unconfirmed positivity seen in the FilmArray™ GI panel may be a consequence of non-specific amplification due to the complex nature of stool specimens and high-order multiplex assays [18]. In addition, the discrepant analyses were performed on thawed samples and thus the quality of viral RNA could be affected.

Overall, and in keeping with the findings of previous studies, 27.2% of the samples were positive for at least two pathogens with a frequent detection incidence of viral and bacterial coinfections [14, 25, 27]. However, the role of coinfections in AGE is still unclear and pathogen associations require further investigation. In this regard, the introduction of quantitative molecular assays could clarify the pathogenetic role or the bystander presence of pathogens associated with GI syndromes. The use of multiplexed PCR such as the FilmArray™ GI panel yielded an increased detection rate for GI pathogens, particularly in mixed infections [24]. This finding has opened up the discussion on the clinical interpretation of these multiple infections and their impact on patient management especially in terms of antimicrobial stewardship.

It is important to mention that this study has several limitations, including the lack of a confirmatory test for certain pathogens and the relatively small number of samples. Due to the limited number of positive samples and the different methods used, we were unable to assess sensitivity and specificity for each pathogen included in the FilmArray™ GI panel. Moreover, the results of this study could be influenced by several factors such as geographic location, season of sampling (December–May) and the patient population analyzed.

## Conclusion

The FilmArray™ GI panel has proved to be a useful tool in the rapid (1 h turnaround time) diagnosis of gastrointestinal pathogens especially in high-risk patients. Due to the increased detection rate and the wide spectrum of diarrheal pathogens detected, the FilmArray™ GI panel has the potential to improve patient management. However, additional studies aimed at evaluating the clinical utility and cost-effectiveness of multiplex molecular testing are needed.

## Abbreviations

AdV: adenovirus
AGE: Acute gastroenteritis
C. *cayetanensis*: *Cyclospora cayetanensis*
*C. difficile*: *Clostridium difficile*
E. *histolytica*: *Entamoeba histolytica*
EAEC: enteroaggregative *E. coli*
EHEC: enterohemorrhagic *E. coli*
EIEC: enteroinvasive *E. coli*
EPEC: enteropathogenic *E. coli*
*ETEC*: enterotoxigenic *E. coli*
FTD: Fast Track Diagnostic.
*G. lamblia*: *Giardia lamblia*
GI: Gastrointestinal
HUS: Hemolytic uremic syndrome
*P. shigelloides*: *Plesiomonas shigelloides*
STEC: Shiga toxin (Stx)–producing *Escherichia coli*
*Y. enterocolitica*: *Yersinia enterocolitica;*
